# Hope

**Published:** 1886-11-27

**Authors:** 


### Words of Consolation.

[Communications for this column should be addressed, "Chaplain," care of the Editor of The Hospital.]

### IX.—Hope.

St. Paul says, "that we through patience might have hope" (Rom. xv, 4), and "Patience worketh experience, and experience hope" (Rom. v, 4). Certainly, next to patience, hope is one of the gifts most essential to the comfort of the sick and suffering. Let us begin by remembering that hope is a gift of God even, as faith and charity. Hope must therefore be prayed for whenever temptations to despair have been allowed an entrance. It must be cherished gratefully by those who possess it as the surest antidote to fall back upon whenever a fit of despondency seizes upon the mind.

Now, although hope is a gift of God, there are some persons born with a great deal more hopefulness in their dispositions than others. It is natural to them to hope for the best in every condition they may happen to occupy. They always look on the brightest side of things, and expect the happiest results. So when they are called to suffer their natural temperament is of the greatest service; they do not easily fall victims to despondency. Others, again, are naturally despondent. They take a gloomy view of life under ordinary circumstances. Much more then in sickness are they likely to take the worst possible view of their own case. These are the patients who must learn to hope even against hope; for "hope deferred maketh the heart sick" (Prov. xiii, 12). But in cultivating a hopeful disposition it does not follow that we must hope merely for recovery from sickness. Heavenly hope reaches much further, and embraces "the things which God hath prepared for them that love Him," and so helps many a sufferer to be bright and cheerful when the prospect of recovery has faded away. Hope for heaven always; but hope must be exercised according to the nature of your case. If the doctors tell you that there is a prospect of recovery, whether that prospect be fair or only a possible one, you are bound to hope for the best. Do not give way to despair. Do not say you are not likely to be any better, or that medicine can be of no use to you, or that if you do get any better, you can never be yourself again. All such feelings are wrong when there is a possibility of recovery; because life is a gift of God, and so long as those who know best say that life is capable of being preserved, it is your duty to think so too, and to assist in its preservation by cherishing hope. The mind acts upon the body. The means, therefore, used for your recovery are far more likely to prove efficacious if you yourself believe in God blessing them, and hope for the best. So, in a certain sense, you may be saved by hope. If, on the other hand, you have been shown plainly that recovery in your case is not to be expected, we still plead for the exercise of the hope which is laid up for you in heaven. Hope in the darkest hours for a very glorious future. Hope for the happiest possible issue out of all your afflictions. Hope, too, for much nearer blessings than such as belong to the Kingdom of Glory. Hope for the more immediate presence of Christ and His Holy Angels; so that whenever you pass through the valley of the shadow of death you may fear no evil, but find even more than you hoped for revealed in the blessed company of the faithful in Paradise.

(*To be continued.*)

